# Health Services and Financing of Treatment

**Published:** 2011

**Authors:** Maureen T. Stewart, Constance M. Horgan

**Keywords:** Alcohol and other drug (AOD) use treatment, treatment costs, health care delivery and administration, health care financing, cost-effectiveness of AOD health services, cost-benefit analysis, health insurance, Medicare, Medicaid, legislation, public policy

## Abstract

Financing, payment, and organization and management of alcohol and other drug (AOD) treatment services are closely intertwined and together determine whether people have access to treatment, how the treatment system is designed, and the quality and cost of treatment services. Since the 1960s, changes in these arrangements have driven changes in the delivery of AOD treatment, and recent developments, including the passage of Federal parity legislation and health reform, as well as increasing use of performance contracting, promise to bring additional changes. This article outlines the current state of the AOD treatment system and highlights implications of these impending changes for access to and quality of AOD treatment services.

## Financing of Alcohol and Other Drug Treatment

Although most general medical services are paid for through private and public insurance mechanisms, insurance coverage has traditionally played a smaller role in provision of alcohol and other drug (AOD) treatment services (Horgan and Merrick 2001). Both private insurance, purchased by employers for their employees, and public insurance, provided by Federal and State governments in the form of Medicare and Medicaid, often have not covered AOD treatment services or severely limited their coverage. In addition, individuals with AOD problems are more likely to be uninsured. That leaves individuals without insurance coverage or with limited insurance coverage for AOD treatment with two options for accessing treatment: paying out of pocket for treatment services or accessing treatment through publicly funded addiction treatment programs.

### Private Financing

Although private insurance spending as a dollar amount has remained stable, it has been declining as a share of total AOD treatment expenditures since 1986, when private insurance contributed $2.8 billion, or almost 30 percent, of all expenditures (Mark et al. 2007*b*). As managed-care organizations began to dominate the private insurance market, extensive utilization management controls effectively eliminated coverage of what had been standard 28-day residential programs and shifted coverage to outpatient care (Shepard and Beaston-Blackman 2002). As a result, private insurance expenditures declined at an average annual rate of 9 percent between 1989 and 1992 and then more slowly at an average annual rate of 3 percent between 1992 and 1998 (Mark et al. 2007*b*). Between 2001 and 2003, private insurance expenditures began to increase at a moderate rate of almost 4 percent per year (Mark et al. 2007*b*), perhaps because of more members accessing services or costlier service mix (e.g., intensive outpatient services displacing outpatient care).

These estimates of private insurance expenditures likely underestimate actual expenditures because they only count AOD problems when they are recorded as the primary diagnosis. It may be that additional AOD treatment is being provided along with other services but is not counted in the estimates. Therefore, private insurance may be covering more AOD treatment than is reflected in these numbers (Mark et al. 2007*b*).

### Public Financing

The public sector funds AOD services in a variety of ways: States contract for services and provide services directly, for example, through the criminal justice system. The Federal Government and State governments pay a share of Medicaid programs. The Federal Government provides insurance coverage through Medicare and provides services directly through the Veteran’s Administration and military facilities.

Together, these public payer programs paid for more than 77 percent of all AOD treatment in 2003 (Mark et al. 2007*b*). In sharp contrast, public payers funded only 45 percent of general health care expenditures (Mark et al. 2007*b*). In addition, because the expenditure estimates for AOD treatment services are calculated as estimates of medical service expenditures only (Mark et al. 2007*a*), they do not represent the full spectrum of services and expenditures, which include wrap-around services such as employment assistance, housing assistance, and transportation services. Therefore, these figures should be thought of as minimum estimates of expenditures for AOD treatment services.

### Public Insurance Financing

The Federal Government and State governments jointly fund the Medicaid program, which covered 14 percent of nonelderly Americans in 2007 (Fronstin 2008). In terms of AOD treatment, Medicaid financed 18 percent of expenditures in 2003, amounting to approximately $3.7 billion. These expenditures grew at an annual rate of 8.5 percent between 1986 and 2003, with a burst of 18.5 percent growth in the early 1990s and slowing to just 5.8 percent between 2001 and 2003, the last 2 years for which data are available. These changes were driven in part by Medicaid expansions as more individuals gained coverage under the program (Kaiser Family Foundation 1998).

Because States operate the Medicaid program and develop their own eligibility requirements and benefit packages within broad Federal guidelines, eligibility and benefits are complex and vary greatly from State to State. A comprehensive review conducted in 1999 identified six States without AOD treatment benefits or with benefits limited to detoxification services (Tompkins and Reif 2006). A more recent study found that 74 percent of 31 States with Medicaid managed-care plans covered outpatient treatment services (Maglione and Ridgely 2006).

### Public Noninsurance Financing

To make up for lean private and public insurance coverage for AOD treatment services, the public sector plays a number of significant roles in purchasing AOD treatment. The figure shows that the public sector, specifically the States, funds the majority of substance use services in the United States—$16.1 billion of the total $20.7 billion spent on AOD disorders in 2003 (Mark et al. 2007*b*).

These funds come from a combination of Federal and State resources. In particular, State and local dollars combine with Federal block grant dollars to fund AOD treatment services for people without adequate public or private insurance who are not able to pay out of pocket. The Substance Abuse Prevention and Treatment (SAPT) Block Grant program was developed in the 1980s to distribute Federal funds to states to purchase AOD treatment services, while limiting the Federal Government’s role and allowing States flexibility to design and purchase services and systems to meet local needs. Congress determines the overall grant amount, which it then allocates to States according to a formula. In 2003, the total Federal block grant was $1.2 billion, or 6 percent of national AOD treatment spending. Between 2001 and 2003, legislative increases in Federal funding raised the Federal block grant at an average annual rate of 4.9 percent.

Although supported by Federal block grant funds, State and local governments pay the largest share of AOD treatment costs, contributing $8.4 billion in 2003 for the direct purchase of services, excluding Medicaid (Mark et al. 2007*b*). From 1993 to 2003 State and local expenditures for AOD treatment services increased at an average annual rate of 6.1 percent per year, whereas total private expenditures remained virtually unchanged (Mark et al. 2007*b*). This resulted in an increase in the State and local share of expenditures from 31 percent in 1993 to 40 percent in 2003 (Mark et al. 2007*b*).

A number of factors likely drove the increase in State and local expenditures, including increases in the number of people mandated to treatment through the criminal justice system (Mark et al. 2007*b*). Indeed, the proportion of admissions with a referral from the criminal justice system increased from 33 percent in 1993 to 38 percent in 2006 (Substance Abuse and Mental Health Services Administration 2009). In addition, as tax revenue increased in many States during a time of expanding economies, States may have allocated additional funds to AOD treatment. Finally, programs that receive most of their funding from the public sector tend to offer more wrap-around services than programs funded primarily by private-sector sources (Ducharme et al. 2007). Although the national spending estimates attempt to remove these nonmedical expenditures, they may not fully succeed. In this case, it may be that expenditures for wrap-around services are included to a greater degree in the estimates for publicly funded treatment services than they are in estimates for programs that are primarily privately funded.

### Client Out-of-Pocket Financing

Client out-of-pocket expenditures increased from $1.2 billion in 1986 to $1.7 billion in 2003 (Mark et al. 2007*b*), an average annual growth rate of 1.5 percent, which is lower than inflation and suggests that client contributions actually declined. Indeed, the share of total expenditures covered by client out-of-pocket payments declined from 14 percent in 1986 to 8 percent in 1989 (see figure) and remained at 8 percent through 2003 (see figure) (Mark et al. 2007*b*). This decline likely can be attributed to increased use of Medicaid, which requires lower copayments, as well as increased use of publicly funded treatment, which requires low or no client cost sharing.

## Payment for AOD Treatment Services

In addition to financing, the design of the payment system under which AOD treatment programs operate influences access to, and quantity and quality of, treatment services. The goal of any payment system is to provide treatment providers with incentives to provide accessible, high-quality treatment.

Historically, both public and private payers have used the same two traditional payment systems for AOD treatment programs: fee for service and fixed budget.

Under the fee-for-service system, payers reimburse programs based on the units of service delivered, which gives programs an incentive to increase the quantity of services and to offer more expensive services. Under the fixed-budget system, payers reimburse programs a fixed amount per year—determined through negotiation and based on past costs—regardless of the number of clients served. Under this payment system, programs have an incentive to limit access and utilization of services because they receive the same payment regardless of the quantity of services delivered.

Both systems lack incentive for provision of quality or cost-effective care, and the provision of a minimum level of service is not an inherent aspect of the fixed-budget system (Horgan and Merrick 2001).

Alternative payment systems, including per-case payments, capitation payments, and performance contracting have been developed to modify the incentives inherent in traditional payment systems. Under a per-case payment system, payers reimburse programs a fixed amount per episode of care, which leaves programs responsible for any costs that exceed the payment. Such a system creates financial incentives to curtail resource use by limiting the intensity and length of stay in treatment. Under a capitated system, payers reimburse programs a fixed amount per enrollee, determined prospectively, for a range of services provided over a period of time, usually 1 year. These programs have an incentive to not only reduce the intensity of service and volume of care but to also shift care from higher- to lower-cost services.

Performance-based contracts (PBCs) have been implemented to try to improve program accountability and provide incentives for high-quality care by tracking measures such as retention in treatment and visit frequency that are thought to be linked to positive patient outcomes. Payers reimburse programs that show improved performance in these activities, or meet specified standards agreed upon in the program’s contract. The amount of money available under the PBCs may be the same, or potentially more, than the base amount available under the original system. In addition, when programs do not meet the measures in the contract they may be penalized, depending on the design of the contract. The measures in the PBC determine program incentives, but generally speaking the PBC provides incentives to programs to deliver the volume and type of services that the purchasers want them to deliver. A number of States, including Delaware, Maine, and Iowa, currently use performance contracts for AOD treatment programs, and Massachusetts and Connecticut are developing, testing, and implementing similar contracts.

An evaluation of Delaware’s switch from a global budget-payment system, which did not require program accountability, to a PBC showed that access to treatment increased dramatically (McLellan et al. 2008). Further analyses showed that waiting time for treatment declined significantly, in part because of the performance contract and in part because of participation in a formal quality-improvement program (Stewart 2009). Results of these studies indicate that access to, and quality of, care improved under the PBC (Stewart 2009).

Regardless of the type of payment system, four specific aspects of payment are critical when evaluating the influence of systems on treatment care (Horgan and Merrick 2001):

- The unit of payment, which, under a traditional payment system, generally is the procedure or service, and under an alternative payment system may be the case (e.g., the entire treatment stay), the episode, or a fixed time period (e.g., a year).
- The method of setting the price, which, under a typical payment system, is based on providers’ costs or the prevailing charge in the area, whereas under an alternative payment system it may be negotiated or set competitively through a bidding process.
- The generosity of payment, which has implications for provider participation in the program. For example, low Medicaid payments have led to a shortage of providers willing to treat Medicaid recipients, thereby increasing waiting time for care and decreasing care quality (Institute of Medicine 2006).
- The organizational level of payment, which determines whether payments go to a program or to individual clinicians within a program. For example, programs may be paid under a performance contract but may in turn pay clinicians on salary. This scenario weakens PBC incentives and, therefore, the PBC may not have the intended effect. Research shows that programs which pass financial incentives on to clinicians have better performance on the PBC (Stewart 2009).

## Organization and Management of AOD Treatment Services

The setting in which AOD treatment services occur, and the services patients receive, are largely determined by what insurance companies and public payers are willing to finance. As of 2007, private health plans provided insurance coverage to 71 percent of nonelderly Americans (Employee Benefit Research Institute [EBRI] 2008). And although a nationally representative survey of private health plans showed that in 2003 most plans offered a range of substance abuse services (Horgan et al. 2009), 81 percent of insured workers were in plans with special limits on substance abuse (Gabel et al. 2007).

In particular, to control costs, these plans frequently place annual limits on the number of inpatient and outpatient AOD treatment services they will cover (Horgan et al. 2009). Specifically, 87 percent of plans limited inpatient care, most commonly using a 30-day annual coverage limit; and 93 percent of plans placed annual limits on outpatient care. The most frequently used restrictions were 20-visit and 30-visit annual limits (Hodgkin et al. 2009). In addition, health plans increasingly require patients to share the cost of AOD treatment services. For example, the proportion of products identified with high cost-sharing requirements for behavioral health care increased from 26 percent in 1999 to 42 percent in 2003 (Horgan et al. 2009). These practices effectively limit access to AOD treatment services for people with private insurance but are likely to change with the implementation of the 2008 Federal Parity Bill, which will be discussed in more detail below.

Another consequence of cost-cutting measures by insurance plans is the push to move from inpatient to outpatient care. As a result, there has been a dramatic increase in the use of specialty substance abuse centers and nonpsychiatric physicians. Between 1998 and 2001, expenditures for specialty substance abuse centers have grown at an average annual rate of 12 percent and continued at 6 percent between 2001 and 2003 (Mark et al. 2007*b*). In addition, expenditures for nonpsychiatric physicians, many of whom may be primary-care physicians, have grown at an average annual rate of 10.2 percent between 1998 and 2001 and almost 5 percent between 2001 and 2003 (Mark et al. 2007*b*).

These numbers coincide with an increased focus on the role of primary-care physicians in identifying, treating, and coordinating AOD treatment services. In 2003, for example, the National Committee for Quality Assurance (NCQA) adopted new performance measures—developed in conjunction with the Washington Circle Group (Garnick et al. 2002)—for identifying, initiating, and engaging in AOD services (NCQA 2003). In that same year, only one-third of plans required behavioral health screening (Horgan et al. 2007). But the new performance measures should change that as health plans work to improve their performance in NCQA’s high-visibility quality-performance system by asking primary-care providers to enhance their screening efforts. In addition, the development of addiction medications makes it increasingly likely that primary-care physicians will be directly involved in treating AOD dependence.

Along with treatment setting, the types of services available often vary based on who is paying for the services. High-quality treatment programs provide services that focus on reducing substance use along with wrap-around services, including transportation assistance, child care, and mental health, employment, and medical services to improve outcomes and retention in treatment. However, not all payers support wrap-around services. Research shows that government-operated programs and publicly funded nonprofit programs offer more wrap-around services than private for-profit programs (Ducharme et al. 2007). Similarly, a small study of programs in Michigan found that programs receiving most of their funding from criminal justice agencies are more likely to offer access to wrap-around services than privately funded programs (Arfken and Kubiak 2009).

Interestingly, the studies mentioned above also found that publicly funded nonprofit programs and programs funded through criminal justice agencies were less likely to offer pharmacotherapy for AOD treatment, despite recommendations by the National Quality Forum that all patients with substance use disorders be assessed for the use of pharmacotherapy as part of their treatment (National Quality Forum 2005). In fact, although the use of medications such as disulfiram, naltrexone, long-acting naltrexone, and acamprosate—which are all available to treat alcohol dependence—is increasing, their use remains relatively low. Indeed, although expenditures for pharmacotherapies increased 17 percent annually from 1998 to 2003, medications to treat AOD use represented just 0.5 percent of AOD treatment expenditures in 2003 (Mark et al. 2007*b*).

## Policy Changes Influencing AOD Treatment Services

Three recent changes indicate that the roles of public and private insurance payers are likely to increase in coming years. First, in 2007, the Centers for Medicare and Medicaid Services (CMS) established payment codes for screening and brief intervention. These codes allow primary-care physicians to bill Medicare, Medicaid, and some private insurance companies for provision of screening and brief intervention counseling. As a result, primary-care physicians may provide more AOD treatment services, which would be covered by insurance companies. Second, the 2008 Federal Parity Legislation, described in more detail below, will require changes to private insurance and Medicaid managed-care coverage, which may expand coverage of AOD treatment services. Finally, the National Health Care Reform legislation requires all individuals to have insurance coverage. This law, particularly in combination with the parity legislation, may result in dramatic changes in access to and delivery of AOD treatment services.

### The 2008 Parity Act

On October 3, 2008, President George W. Bush signed into law the Paul Wellstone and Pete Domenici Mental Health Parity and Addiction Equity Act of 2008 as part of the Emergency Economic Stabilization Act of 2008. This Federal legislation aimed to remove barriers to utilization, remove financial burdens on patients, and reduce stigma around addictive and mental disorders by requiring group health plans that offer mental health/addiction services to cover these services in a comparable manner to medical/surgical services.

The new law, which expands a more limited 1996 Federal law that did not apply to substance abuse treatment, applies to all health plans, including self-insured and Medicaid managed-care plans, but exempts group health plans of fewer than 50 employees. Effective on January 1, 2010, the new law requires “equity in coverage,” meaning that any mental health and addictions benefits that a plan offers must be comparable to medical and surgical benefits. This is applicable to all financial requirements such as deductibles, copayments, coinsurance, and out-of-pocket expenses, and to all nonfinancial treatment limitations, such as frequency of treatment, number of visits, days of coverage, processes for continuing review, and determination of medical necessity.

Whereas the legislation requires parity between medical/surgical benefits and mental health/addiction benefits if a plan offers these benefits, it does not mandate that plans cover mental health/addiction treatment. Also, it does not mandate that programs cover all mental health/addiction conditions; plans can exclude specific diagnoses from coverage. Indeed, there is a concern that companies that had previously offered some benefits for mental health and addiction services might choose to drop coverage in response to parity legislation.

### Health Reform

The Patient Protection and Affordable Care Act, signed into law by President Barack Obama in March, 2010, requires all Americans to have health insurance. This law will increase insurance access by expanding Medicaid eligibility and mandating individual insurance coverage. The Medicaid eligibility expansion will result in increased access to substance abuse treatment coverage through Medicaid. The individual insurance expansion also may result in improved access for those with private insurance, but private insurance companies are not required to cover AOD treatment services. AOD treatment programs that provide services for public clients will likely experience a shift in payer from block grant funds to Medicaid funds.

The National Association of State Alcohol and Drug Abuse Directors (NASADAD) conducted case studies of three States—Maine, Massachusetts, and Vermont—that previously enacted health reform. The results provide some indication of how Federal health reform likely will affect access to and quality of AOD treatment services (NASADAD 2010). Although State health reform initiatives expanded insurance coverage, NASADAD found that block grant funding remained important for financing wrap-around services, residential treatment, and prevention efforts. Despite these limitations, people living in all three States had increased access to AOD treatment services and the States achieved cost savings through the use of prior authorization requirements. Under prior authorization requirements, services will not be covered unless providers or patients first obtain approval from insurance companies. These requirements may not be allowed under recently passed Federal parity legislation, so it is not clear that cost savings will continue to be achieved.

## Conclusion

The Federal Parity Law, in combination with national health care reform has the potential to transform delivery of behavioral health services. The Federal Parity Law may lead to increased private and public insurance financing for some types of substance abuse treatment. In addition, the parity law is likely to result in changes to the management of AOD treatment services under private and public insurance as insurers will have to apply similar processes to medical and behavioral health care. National health care reform is likely to bring additional change to the sector as access to insurance coverage expands and may lead to increased access to substance abuse treatment services. As the financing and management of substance abuse treatment services evolve, it will be important to understand how these changes effect access to and quality of substance abuse treatment services.

## Figures and Tables

**Figure:**
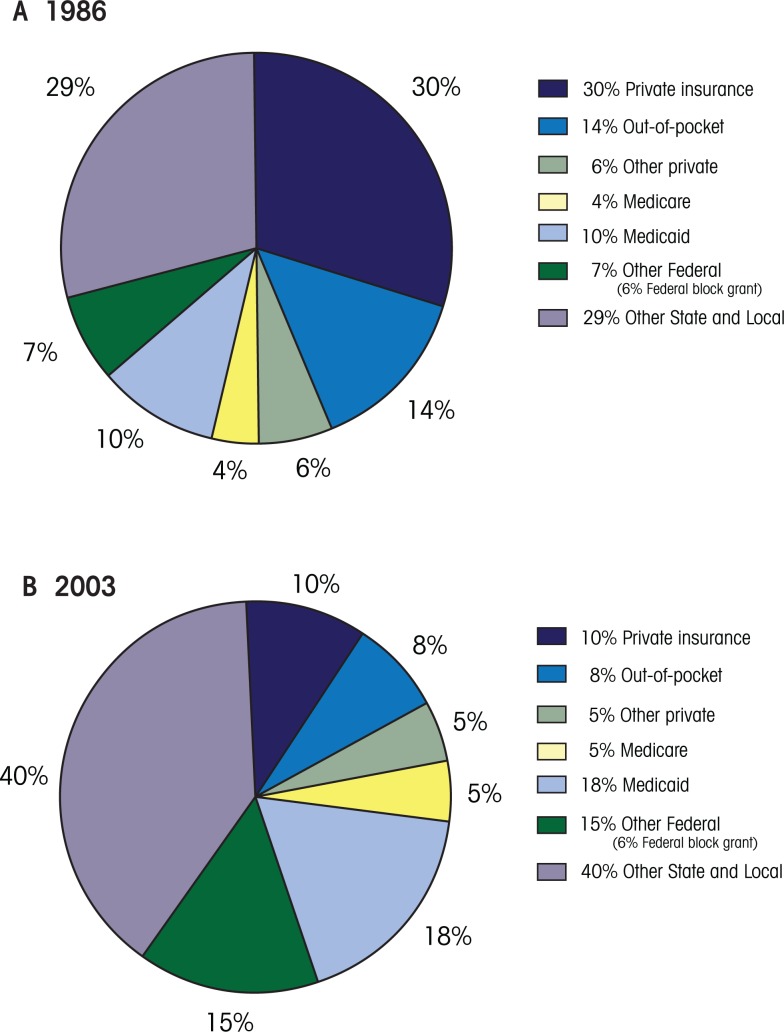
Distribution of funding of AOD treatment by payer for 1986 **(A)** and 2003 **(B)**. SOURCE: Mark, T.L.; Coffey, R.M.;, McKusick, D.R.; et al. *National Expenditures for Mental Health Services and Substance Abuse Treatment,* 1993–2003. Rockville, MD: Substance Abuse and Mental Health Services Administration, 2007.
